# Supplementary material for: Spectral cross-cumulants for multicolor super-resolved SOFI imaging
Source: Nat Commun. 2020 Jun 15;11:3023. doi: 10.1038/s41467-020-16841-1 (PMC7295763; doi:10.1038/s41467-020-16841-1)
Supplement: Supplementary file 1 — Supplementary Information [file 41467_2020_16841_MOESM1_ESM.pdf]

Supplementary Information

Spectral Cross-Cumulants for Multicolor Super-resolved SOFI Imaging

K. S. Grußmayer[1,2,#,*], S. Geissbuehler[2,#], A. Descloux[1,2], T. Lukes[1,2], M. Leutenegger[2,3], A. Radenovic[1], T. Lasser[2,4,*]

Affiliations

[1]École Polytechnique Fédérale de Lausanne, Laboratory of Nanoscale Biology, 1015 Lausanne, Switzerland

[2]École Polytechnique Fédérale de Lausanne, Laboratoire d'Optique Biomédicale, 1015 Lausanne, Switzerland

[3]Max-Planck Institute for Biophysical Chemistry, Department of NanoBiophotonics, Am Fassberg 11, 37077 Göttingen, Germany

[4]Max-Planck Institute for Polymer Research, Ackermannweg 10, 55128 Mainz, Germany

Contributions

[#]These authors contributed equally to this work.

Corresponding Author:

Theo Lasser

* email: kristin.grussmayer@epfl.ch, theo.lasser@epfl.ch

Supplementary Note 1: Theory of spectral unmixing using spectral cross-cumulants

n^th^ order spectral cross-cumulant analysis between two adjacent physical spectral channels

For the case of 2 physical spectral channels (here: transmission channel T and reflection channel R, collecting fluorescence light in a specific wavelength range. A according to the combined spectral response of all the filters implemented in the microscope, with corresponding transmission T_i and reflection coefficients R_i per fluorophore species *i*) and a total of N_c fluorophore species we write:

$$\begin{aligned} I_{\mathrm{R}}(\boldsymbol{r}) &= \sum_{i=1}^{N_{\mathrm{c}}} R_i I_i(\boldsymbol{r}) \\ I_T(\boldsymbol{r}) &= \sum_{i=1}^{N_{\mathrm{c}}} T_i I_i(\boldsymbol{r}) \end{aligned} \tag{S1}$$

with $I_i(\boldsymbol{r})$ being the intensity distribution of species *i* measured on detector pixel $\boldsymbol{r}$. This linear system cannot be inverted to solve for the images of the isolated fluorophore species for more unknowns $I_1(\boldsymbol{r})$, $I_2(\boldsymbol{r})$, …, $I_{N_{\mathrm{c}}}(\boldsymbol{r})$ than measurements, i.e. for$N_{\mathrm{c}} > 2$ in this case.

However, if we assume stochastic, independent blinking of all the fluorescent emitters of the different species[1], we can apply cumulant analysis on the time series recorded in the transmission and reflection channels and generate an additional $n-1$ virtual channels by computing the nth-order cross-cumulants (provided appropriate sampling of the PSFs). Due to the additivity property, the cumulant of multiple independent species corresponds to the sum of the cumulants of each individual species and we can rewrite:

$$\kappa_{\mathrm{n,(T,\dots,R)}}(\boldsymbol{r}) = \sum_{i=1}^{N_{\mathrm{c}}} T_i^u R_i^{n-u} \kappa_{\mathrm{n}}\{I_i(\boldsymbol{r},t)\} \tag{S2}$$

With (T, … , R) the set of *n* physical channels denoting the cross-cumulant that is computed using *u* pixels from the transmission channel and $n-u$ pixels from the reflection channel. $\kappa_n\,\{I_i(\boldsymbol{r},t)\} = \kappa_{n;i}$ denotes the nth-order cumulant of the different fluorophore species *i*.

General case of nth-order spectral cross-cumulant analysis and N_p physical color channels

For the most general case of N_{p} physical spectral channels (psc) and N_{c} fluorescent species *i*, we can define a corresponding proportion $P_{psc,i}$ of the intensity that is directed into the specific spectral channel.

$$I_{psc}(\boldsymbol{r}) = \sum_{i=1}^{N_{\mathrm{c}}} P_{\mathrm{psc},i} I_i(\boldsymbol{r}) \tag{S3}$$

If we assume stochastic, independent blinking of all the fluorescent emitters of the different species, we can again generate virtual channels by computing the nth-order cross-cumulants. Due to the additivity, the cumulant of multiple independent species corresponds to the sum of the cumulants of each individual species and we can rewrite:

$$\kappa_{\mathrm{n,(psc_1,\ldots,psc_n)}}(\boldsymbol{r}) = \sum_{i=1}^{N_{\mathrm{c}}} \left(\prod_{j=1}^{n} P_{\mathrm{psc_j},i} \right) \kappa_n \ \{I_i(\boldsymbol{r}, t)\} \tag{S4}$$

With $(psc_1, \ldots, psc_n)$ the set of physical spectral channels $psc_j \in \{1, \ldots, N_{\mathrm{p}}\}$ denoting the cross-cumulant that is computed using pixels from the physical spectral channel psc_j. $\kappa_n \ \{I_i(\boldsymbol{r}, t)\} = \kappa_{n;i}$ denotes the nth-order cumulant of the different fluorophore species *i*. This computation of additional channels is the key to enable unmixing by inversion of the linear system of equations.

Single-color single-species cumulant for m emitters:

For *m* fluorescent emitters of a single fluorophore species *i* recorded in a single color channel, the nth-order cumulant can be written as

$$\kappa_{n,i}(\boldsymbol{r}) \propto \ \varepsilon_i^n(\boldsymbol{r}) f_{n,i}\big(\rho_{\mathrm{on},i}; \boldsymbol{r}\big) \sum_{k=1}^{m} U_i^n(\boldsymbol{r} - \boldsymbol{r}_k), \tag{S5}$$

where $\varepsilon_i(\boldsymbol{r})$ is the the spatial distribution of the molecular brightness, $f_{n,i}(\rho_{\mathrm{on},i};\boldsymbol{r})$ is the nth-order cumulant of a Bernoulli distribution with on-time ratio $\rho_{\mathrm{on},i} = \frac{\tau_{\mathrm{on},i}}{\tau_{\mathrm{on},i}+\tau_{\mathrm{off},i}}$ and $U_i(\boldsymbol{r})$ is the system PSF for the fluorophore species *i*[2].

Flattening:

Using cross cumulants, virtual pixels are calculated in between the physical pixels acquired by the camera. Subsequently, proper weights are assigned to these virtual pixels in the so-called flattening operation assuming a known PSF[3] (see Equation S6) or optimal weights are calculated using a computationally demanding approach based on jackknife resampling[4].

$$\chi_{n;i}\left(\boldsymbol{r} = \frac{1}{n}\sum_{k=1}^{n}\boldsymbol{r}_k\right) = \frac{\kappa_{n;i}(\boldsymbol{r}_1, \ldots, \boldsymbol{r}_n)}{d_{n,k}(\boldsymbol{r}_1, \ldots, \boldsymbol{r}_n)} \tag{S6}$$

Where $d_{n,k}(\boldsymbol{r}_1, \ldots, \boldsymbol{r}_n) = \prod_{j<l}^{n} U_i\left(\frac{\boldsymbol{r}_j - \boldsymbol{r}_k}{\sqrt{n}}\right)$ is the distance factor.

In this study, we used the simple yet effective approach of weighing to the same mean within sub-grids of the image. Calculating the nth order cross-cumulant, we obtain *n-1* virtual pixels in between each pair of pixels of the original pixel grid. For example, in the case of the 2nd order cumulant, we generate 3 virtual pixels for each physical pixel (i.e. there are 4 "pixel types" in the new, finer grid). This new grid can be divided into 4 mutually shifted sub-grids (each composed out of pixels of the same "pixel type"). These sub-grids represent the same image shifted by p_s/n, where p_s is the projected pixel size of the original image and *n* is the cumulant order. Assuming that these mutually shifted subsampled versions of the full image are supposed to have the same mean, the flattening can be performed by simply normalizing the sub-grids to the same mean value as the mean of the original image (i.e. sub-grid composed of the physical pixels).

Supplementary Note 2: Transmission and reflection coefficients

From spectral data of the used fluorophores and filters

The known fluorophore emission spectra are weighted with the spectral response curves of the reflection and transmission channels obtained from transmission data of the different (dichroic) filters that are implemented in the microscope (see *Microscope setup* section in Methods of the main text). An example of the fluorophore Alexa Fluor 568 and the dichroic splitting ~ 594nm is provided in Supplementary Figure 1.

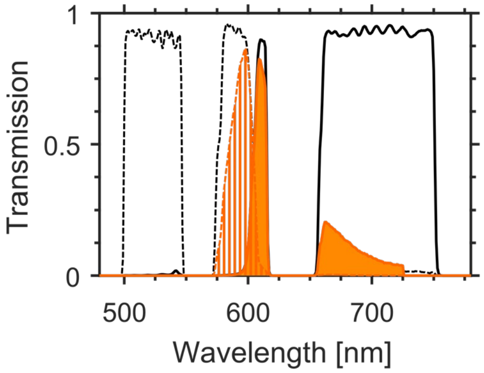

Supplementary Figure 1 Emission spectra of Alexa Fluor 568 multiplied with the spectral response curve of the reflection and transmission channel obtained by a dichroic color splitter (~594 nm) and a multi-band dichroic and emission filter to suppress the excitation laser light. Alexa Fluor 568 emission in the reflection channel (dark orange stripes), Alexa Fluor 568 emission in the transmission channel (dark orange), spectral response of the reflection channel (black dashed line) and of the transmission channel (black solid line).

Here, we assume $D_T(\lambda) + D_R(\lambda) = 1$ and use the transmission data provided by the manufacturer (can also be measured in a spectrometer). Subsequently, the transmission T_i and reflection coefficients R_i per fluorophore species *i* can be calculated by integrating the transmitted (see Supplementary Figure 1 dark orange) and reflected emission spectra (see Supplementary Figure 1 dark orange stripes), respectively, and by normalizing with the total emission: $R, T = \frac{\int_0^\infty S_i(\lambda) D_{T,R}(\lambda) d\lambda}{\int_0^\infty S_i(\lambda) d\lambda} = \frac{I_{R,T}}{\sum(I_R + I_T)}$. The transmission coefficients for the fluorophore and filter combinations used in this work are provided in Supplementary Supplementary Table 1.

Fluorophore	***Dichroic mirror splitting fluorescence emission***	***Transmission coefficient T***
Alexa Fluor 488	Laser Beamsplitter zt 594 RDC	*0.02*
Alexa Fluor 488	Beamsplitter HC BS 640 imaging	*0.04*
Dreiklang	Laser Beamsplitter zt 594 RDC	*0.01*
Janelia Fluor 549	Laser Beamsplitter zt 594 RDC	*0.16*
Atto 565	Laser Beamsplitter zt 594 RDC	*0.35*
Atto 565	Beamsplitter HC BS 640 imaging	*0.18*
Abberior Flip 565	Laser Beamsplitter zt 594 RDC	*0.26*
Alexa Fluor 568	Laser Beamsplitter zt 594 RDC	*0.47*
Alexa Fluor 647	Laser Beamsplitter zt 594 RDC	*0.98*
Alexa Fluor 647	Beamsplitter HC BS 640 imaging	*0.97*
Mitotracker Deep Red FM	Laser Beamsplitter zt 594 RDC	*0.98*

Supplementary Table 1 Transmission coefficients calculated according to the spectral response of the transmission channel for different fluorophores and dichroic mirrors in the emission path.

Experimental determination

Cells are labelled with a single fluorophore species *i* and widefield images across the same filter combination as in the multicolour experiments are obtained in the reflection and emission channel. Subsequently, the transmission $T_i$and reflection coefficients R_i for fluorophore species *i* can be calculated by summing the background corrected transmitted and reflected intensity, respectively, and normalizing by the total emission.

Supplementary Note 3: Optimization of multicolor SOFI via eigenvalue and eigenvector analysis

The selection of the best combination of dyes and adequate filter sets can be an overwhelming and challenging task. In this section, we discuss a systematic approach to guide potential users in this process.

Each fluorophore is characterized by its emission spectrum $S_i(\lambda)$. If we assume that we use a perfect dichroic filter with a transmission function

$$D_T(\lambda; \lambda_D) = H(\lambda - \lambda_D)$$

where $H(\lambda)$ is the Heaviside step function and λ_D is the characteristic wavelength of the dichroic, we can express the transmission and reflection coefficients R_i, T_i as

$$T_i = \int_0^{\lambda_D} S_i(\lambda) d\lambda$$

$$R_i = \int_{\lambda_D}^{\infty} S_i(\lambda) d\lambda$$

where we assume that the spectrum $S_i(\lambda)$ is already normalized. Similarly, we can express the unmixing matrix M as a function of λ_D

$$M(\lambda_D) = \begin{pmatrix} R_1^2 & R_2^2 & R_3^2 \\ T_1 R_1 & T_2 R_2 & T_3 R_3 \\ T_1^2 & T_2^2 & T_3^2 \end{pmatrix} = \begin{pmatrix} \left(\int_{\lambda_D}^{\infty} S_1(\lambda) d\lambda\right)^2 & \left(\int_{\lambda_D}^{\infty} S_2(\lambda) d\lambda\right)^2 & \dots \\ \left(\int_{\lambda_D}^{\infty} S_1(\lambda) d\lambda\right)\left(\int_0^{\lambda_D} S_1(\lambda) d\lambda\right) & \dots & \dots \\ \left(\int_0^{\lambda_D} S_1(\lambda) d\lambda\right)^2 & \dots & \dots \end{pmatrix}$$

The matrix $M(\lambda_D)$ is not invertible if one or more of its eigenvalues are equal to 0. In practice, the linear system is degraded by several noise sources and a matrix with an eigenvalue close to 0 is likely to be unstable. In order words, we need to optimize the product $|\lambda_1||\lambda_2||\lambda_3|$ or maximize the smallest eigenvalue. Using $M(\lambda_D)$ we can compute the eigenvalues λ_i for any set of fluorophores as a function of λ_D.Supplementary Figure 2 a shows the spectal response of three dyes Alexa Fluor 488, Atto 565, Alexa

Fluor 647 and the filter set of the microscope. Supplementary Figure 2 b displays the absolute value of the three eigenvalues of $M(\lambda_D)$ as a function of λ_D as well as their product. As expected, when λ_D is smaller than 560nm or greater than 700nm, at least one eigenvalue tends to 0, meaning that at least two dyes are completely reflected or transmitted. We observe a maximum for the eigenvalues product at around 600nm, which corresponds to the theoretical optimal splitting wavelength.

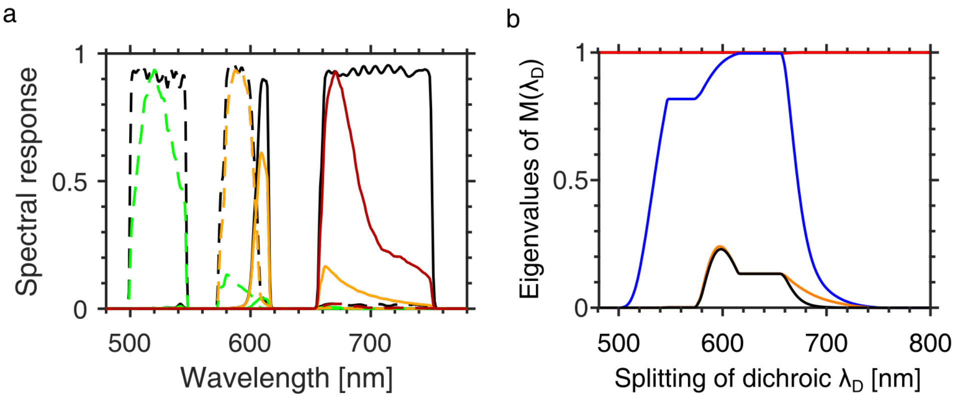

Supplementary Figure 2 Theoretical analysis of dyes-dichroic filter combinations. (a) Emission spectra of common organic fluorophores multiplied with the spectral response curve of the reflection and transmission channel obtained by a dichroic color splitter (~594 nm) and a multi-band dichroic and emission filter to suppress the excitation laser light. Alexa Fluor 488 (green), Atto565 (yellow), Alexa Fluor 647 (red), spectral response of the reflection channel (black dashed line) and of the transmission channel (black solid line). (b) Eigenvalues (red, blue and orange lines) of $M(\lambda_D)$ for $\lambda_D \in [450; 800nm]$ and product of the three eigenvalues (black line) with a maximum at 600nm indicating the theoretical best splitting ratio for this choice of dyes.

In the case of a real dichroic with non-idealized reflection and transmission characteristics, we have to rewrite R and T as

$$T_{i,j} = \int_0^\infty S_i(\lambda) D_j(\lambda) d\lambda$$

$$R_{i,j} = \int_0^\infty S_i(\lambda) \left(1 - D_j(\lambda)\right) d\lambda$$

We can then rank any dyes and dichroic combination and select the $S_1(\lambda)$, $S_2(\lambda)$, $S_3(\lambda)$ and $D_j(\lambda)$ that produces the least singular matrix. In our case, the choice of ZT594RDC with a splitting at 594nm results in the eigenvalues: 1.05, 1.05 and 0.245 (product of 0.27), validating the choice of the dichroic for this specific set of dyes.

The generalization to more channels and dyes is straightforward and will just add additional vectorial components to this eigenvalue/vector analysis.

The unmixing matrix $M(\lambda_D)$ corresponding to the experiments and simulations in this work is diagonalized $M = Q\Lambda Q^{-1}$ with $Q = [v_1\ v_2 v_3]$ and $\Lambda_{ii} = \lambda_i$, as the three eigenvectors form a basis. As $M(\lambda_D)$ needs to be invertible, we choose λ_D such that $\lambda_i \neq 0$ (see discussion above). Thus, the dimension of the image of M is the same as the dimension of its domain and the rank of the unmixing matrix equals the number of color channels. This confirms that our cumulant analysis indeed provides an independent third channel allowing the unmixing of the three fluorophore species.

Supplementary Note 4: Co-registration of physical color channels

Co-registration based on calibration measurements with beads

An affine transformation and bilinear interpolation (Matlab) based on a calibration measurement with fluorescent beads that can be detected in both physical channels is applied to the transmission channel. We typically use ∅ 0.2 µm TetraSpeck beads or ∅ 0.17 µm orange beads from the PSF calibration kit (Invitrogen) dried on glass and covered in the supplied immersion medium. For the measurements presented in the paper the co-registration precision (vector sum of coordinate displacement in original vs. co-registered channel) is ~ 10-30 nm. The virtual spectral cross-cumulant channel has by construction half the registration error with respect to the physical color channels. The unmixing step is a linear operation, thus the registration between the final unmixed color channels is comparable to the co-registration precision of the physical color channels - an order of magnitude better than the attainable resolution for second order analysis. It is noteworthy that careful co-registration is important for cross-cumulant analysis, but sufficient accuracy is routinely achieved.

Co-registration based on image correlation

The temporal standard deviation of the transmission and reflection channels is computed to generate two background free images. An affine transformation and bilinear interpolation (Matlab) based on the normalized cross-correlation between the two images is then applied to all the frames of the transmission channel.

Supplementary Note 5: Simulations - multicolor SOFI with spectral unmixing

To this end, we investigated the influence of different photophysical properties of the fluorophores on the performance of our multicolor analysis in simulations (for details on the simulations, please consult the main text and the Methods section). We simulate thin, densely labelled filaments that are partially overlapping, mimicking the cytoskeleton of cells. We first confirm multicolour imaging with fluorophores that range from green to (far infra-) red emission and verify that different photobleaching and blinking kinetics do not impair multicolour imaging as long as cumulant analysis is appropriately performed. Last, we show that our concept is also able to discriminate between three fluorophores with largely overlapping emission spectra.

We simulated filaments (dimensions ~ 5 µm × 0.04 µm) labelled with the commonly used fluorophores Alexa Fluor 488, Atto 565 and Alexa Fluor 647 (see Supplementary Figure 2). We chose the photophysical parameters to resemble typical SOFI conditions (see Ref. [5] and Supplementary Figure 3) of densely labelled structures with blinking, but multiple overlapping emitters. Multicolour analysis is performed using coregistration based on simulated calibration measurements with multicolour beads that appear 50:50 in both physical color channels. We can faithfully recover the appropriate color channels, as can be seen in Supplementary Figure 4c-f. We estimated the residual crosstalk between the recovered channels by quantifying the remaining background corrected signal of the non-overlapping part of the filaments (see Supplementary Table 2). The estimated crosstalk of Alexa Fluor 488 into the Atto 565 channel was highest and with only 5 % still very low. All other contributions from fluorophores to the “wrong” channels were smaller. We could not determine the residual crosstalk in experiments, as a sample with non-overlapping structures and the fluorophores in question was not available (note: a technical sample with entirely separate structures would be best). An extremely faint spot at the crossing of the Alexa Fluor 488 with the Alexa Fluor 647 filament is seen in the unmixed Atto 565 channel, most likely due to spurious

correlation arising from the very dense blinking with a high on-ratio. For these densely labelled structures with high on-ratio blinking it suffices to analyse only a few hundred simulated frames (see Supplementary Figure 5). This allows extremely fast and easy-to-implement multicolour imaging.

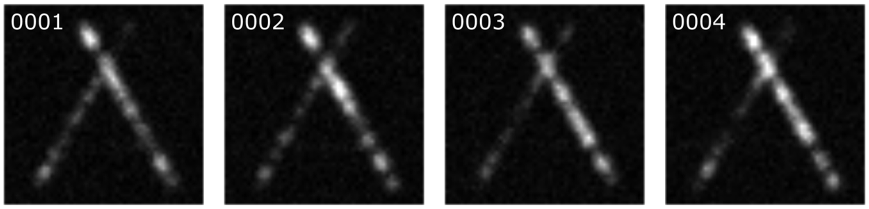

Supplementary Figure 3 Blinking of dense emitters with high on-ratio. Simulations of three fluorophores with emission in the green to (near infra-) red range. Alexa Fluor 488 (horizontal filament), Atto 565 (left, inclined to the right) and Alexa Fluor 647 (right, inclined to the left) at a density of 1000 fluorophores µm^{-2} for a 48 pixel × 0.4 pixel filament (corresponding to 5.184 µm × ~43 nm), I_{on} = 400 photons, on-ratio ρ_{on} = 0.1 (τ_{on} = 20 ms, τ_{off} = 180 ms) and τ_{PB} = 80 s. Four raw frames from the transmission channel illustrate the simulated photophysics.

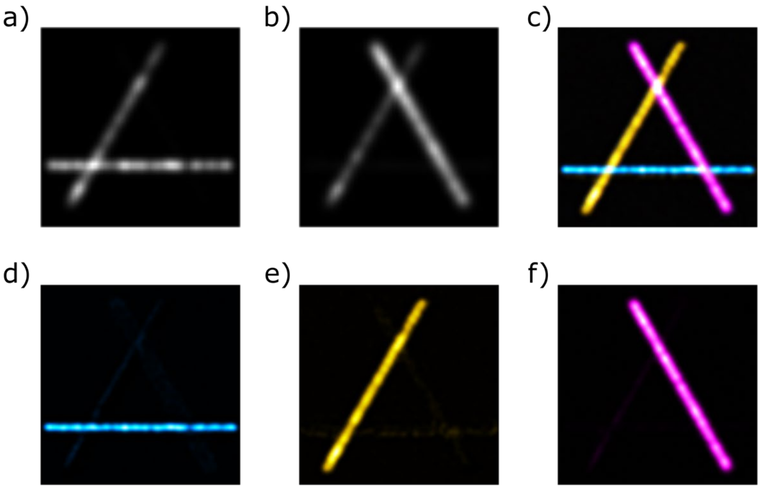

Supplementary Figure 4 Simulations of three fluorophores with emission in the green to (near infra-) red range. Alexa Fluor 488 (horizontal filament), Atto 565 (left, inclined to the right) and Alexa Fluor 647 (right, inclined to the left) at a density of 1000 fluorophores µm^{-2} for a 48 pixel × 0.4 pixel filament (corresponding to 5.184 µm × ~43 nm), I_{on} = 400 photons, on-ratio ρ_{on} = 0.1 (τ_{on} = 20 ms, τ_{off} = 180 ms) and τ_{PB} = 80 s. a) and b) Average of 4000 frames in the reflection and transmission channel. c) Composite image of the balanced second-order SOFI images with d) Alexa Fluor 488 (cyan hot), e) Atto 565 (yellow hot) and f) Alexa Fluor 647 (magenta hot).

Fluorophore/channel	Alexa Fluor 488	Atto 565	Alexa Fluor 647
**Alexa Fluor 488**	100	5	1
**Atto 565**	2	100	2
**Alexa Fluor 647**	2	4	100

Supplementary Table 2 Relative crosstalk in % determined in the three-color simulated sample in Supplementary Figure 4 with the fluorophores Alexa Fluor 488, Atto 565 and Alexa Fluor 647, when only background corrected pixels from regions without filament overlap were considered. The table is read line-wise with the fluorophore whose signal bleeds through listed on the left (e.g. read as: 2 % of Atto 565 bleeding into the unmixed Alexa Fluor 488 channel).

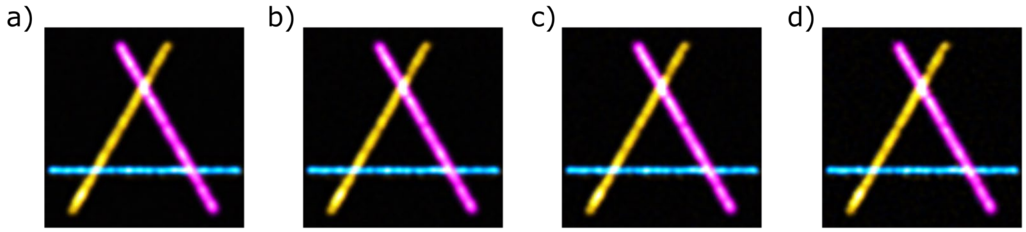

Supplementary Figure 5 Simulations of three fluorophores with varying number of frames used for analysis. Alexa Fluor 488 (horizontal filament), Atto 565 (left, inclined to the right) and Alexa Fluor 647 (right, inclined to the left) at a density of 1000 fluorophores μm^{-2} for a 48 pixel × 0.4 pixel filament (corresponding to 5.184 µm × ~43 nm), I_{on} = 400 photons, on-ratio ρ_{on} = 0.1 (τ_{on} = 20 ms, τ_{off} = 180 ms) and τ_{PB} = 40 s. Composite image of the balanced second-order SOFI images with Alexa Fluor 488 (cyan hot), Atto 565 (yellow hot) and Alexa Fluor 647 (magenta hot) with a) 4000, b) 2000, c) 1000 and d) 500 frames used for analysis.

Fluorophores with different photobleaching kinetics

To study the influence of differences in photobleaching, we considered Alexa Fluor 488, Atto 565 and Alexa Fluor 647 as above and only change the photobleaching time to 10, 40 and 80 s, respectively (see Supplementary Figure 6). This already covers almost one order of magnitude difference in photostability. There is no noticeable change in the performance of our analysis, as the blinking kinetics and cumulant analysis are still appropriate for the fluorophore with the worst stability.

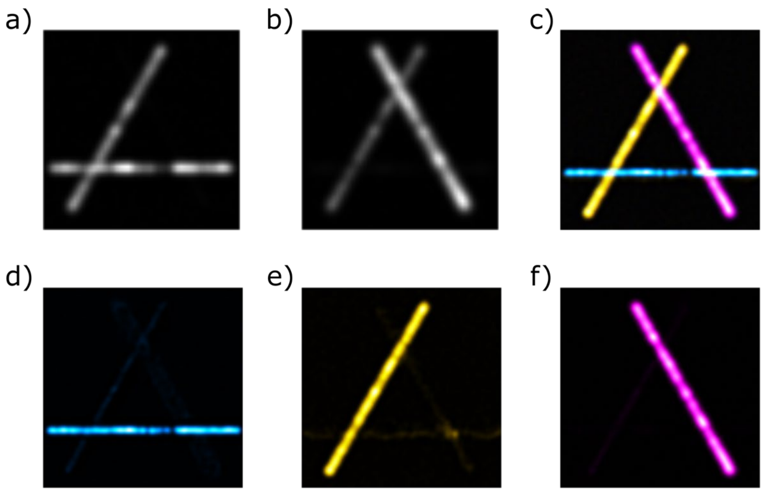

Supplementary Figure 6 Simulations of three fluorophores with different photobleaching kinetics. Alexa Fluor 488 (horizontal filament) with τ_{PB} = 10 s, Atto 565 (left, inclined to the right) with τ_{PB} = 40 s and Alexa Fluor 647 (right, inclined to the left) with τ_{PB} = 80 s at a density of ~1000 fluorophores µm^{-2} for a 48 pixel × 0.4 pixel filament (corresponding to 5.184 µm × ~43 nm), I_{on} = 400 photons, on-ratio ρ_{on} = 0.1 (τ_{on} = 20 ms, τ_{off} = 180 ms). a) and b) Average of 4000 frames in the reflection and transmission channel. c) Composite image of the balanced second-order SOFI images with d) Alexa Fluor 488 (cyan hot), e) Atto565 (yellow hot) and f) Alexa Fluor 647 (magenta hot).

Fluorophores with different blinking kinetics

Since not all fluorophores show the same blinking performance under identical experimental conditions[6], we tested the algorithm with different blinking off-times. As in the first simulations above, we considered Alexa Fluor 488, Atto 565 and Alexa Fluor 647 and only changed the on-ratio to 0.05, 0.1 and 0.01 (τ_{on} = 20 ms, τ_{off} = 380 ms, 180 ms and 1980 ms), respectively. This covers one order of magnitude difference in off-switching kinetics. The algorithm recovers all three color channels faithfully (see Supplementary Figure 7).

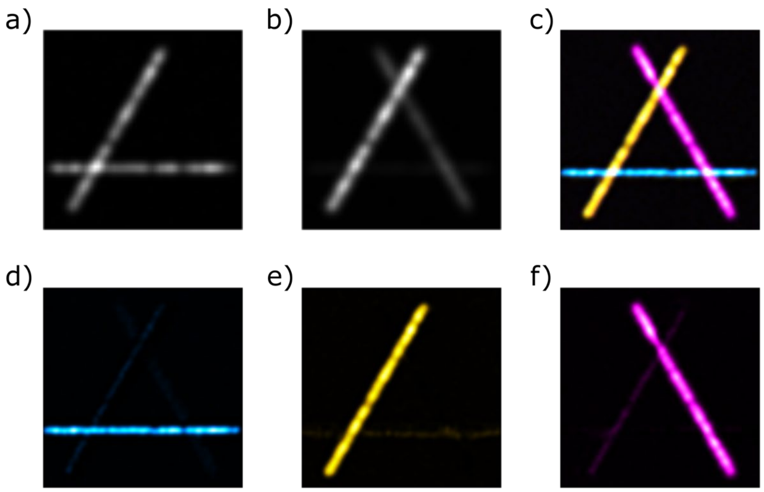

Supplementary Figure 7 Simulations of three fluorophores with different blinking kinetics. Alexa Fluor 488 (horizontal filament) with on-ratio ρ_{on} = 0.05 (τ_{on} = 20 ms, τ_{off} = 380 ms), Atto 565 (left, inclined to the right) with on-ratio ρ_{on} = 0.1 (τ_{on} = 20 ms, τ_{off} = 180 ms) and Alexa Fluor 647 (right, inclined to the left) with on-ratio ρ_{on} = 0.01 (τ_{on} = 20 ms, τ_{off} = 1980 ms) at a density of ~1000 fluorophores μm^{-2} for a 48 pixel × 0.4 pixel filament (corresponding to 5.184 µm × ~43 nm), I_{on} = 400 photons and τ_{PB} = 80 s. a) and b) Average of 4000 frames in the reflection and transmission channel. c) Composite image of the balanced second-order SOFI images with d) Alexa Fluor 488 (cyan hot), e) Atto 565 (yellow hot) and f) Alexa Fluor 647 (magenta hot).

Fluorophores with different brightness

Similarly, it is difficult to achieve equal brightness for all fluorophores in experiments due to different spectral properties and blinking behaviour. As in the first simulations above, we considered Alexa Fluor 488, Atto 565 and Alexa Fluor 647 and only changed the intensity from I_{on} = 400, 600 to 800 photons (τ_{on} = 20 ms, τ_{off} = 180 ms), respectively. The algorithm recovers all three color channels faithfully (see Supplementary Figure 8) and the reconstructed filaments have no obvious difference in contrast.

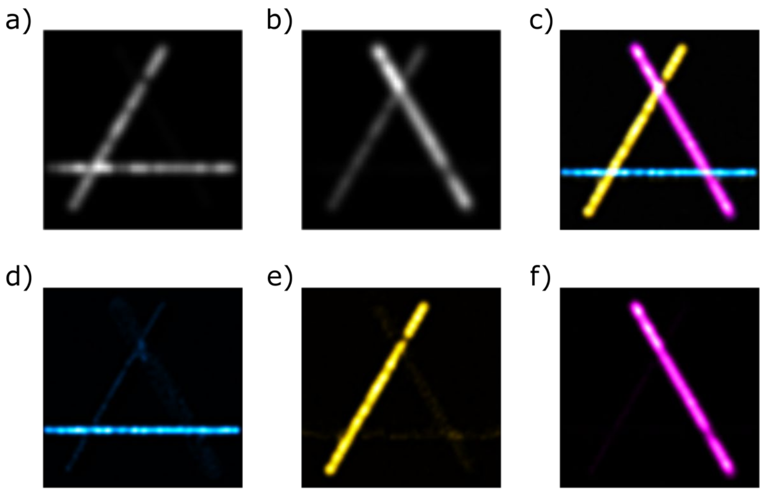

Supplementary Figure 8 Simulations of three fluorophores with different intensity. Alexa Fluor 488 (horizontal filament) I_{on} = 400 photons, Atto 565 (left, inclined to the right) with I_{on} = 600 photons and Alexa Fluor 647 (right, inclined to the left) with I_{on} = 800 photons (τ_{on} = 20 ms, τ_{off} = 180 ms) at a density of ~1000 fluorophores µm^{-2} for a 48 pixel × 0.4 pixel filament (corresponding to 5.184 µm × ~43 nm) and τ_{PB} = 80 s. a) and b) Average of 4000 frames in the reflection and transmission channel. c) Composite image of the balanced second-order SOFI images with d) Alexa Fluor 488 (cyan hot), e) Atto 565 (yellow hot) and f) Alexa Fluor 647 (magenta hot).

Fluorophores with largely overlapping spectra

Next, we changed only the spectral parameters and simulated three fluorophores with largely overlapping emission spectra separated by about 10 nm only, such that the fluorophores can all be excited by one laser line (here: e.g. 561nm for Abberior Flip 565 ($\lambda_{\frac{\text{abs}}{\text{em}},\text{max}} = 566/\,580\text{ nm}$), Atto 565 ($\lambda_{\frac{\text{abs}}{\text{em}},\text{max}} = 564/\,590\text{ nm}$) and Alexa Fluor 568 ($\lambda_{\frac{\text{abs}}{\text{em}},\text{max}} = 578/\,603\text{ nm}$), see Supplementary Figure 9). The multicolour results in Supplementary Figure 10 show that our algorithm can even separate these fluorophores that are impossible to distinguish from the average diffraction limited reflection and transmission images. A detailed inspection of the images shows faint ghost images of filaments from the other channels. We again calculated the residual crosstalk between the recovered channels by quantifying the remaining background corrected signal of the non-overlapping part of the filaments (see Supplementary Table 3). The estimated crosstalk of Alexa Fluor 568 and Abberior Flip 565 into the Atto

565 channel was highest with 14%. All other contributions from fluorophores to the “wrong” channels were smaller.

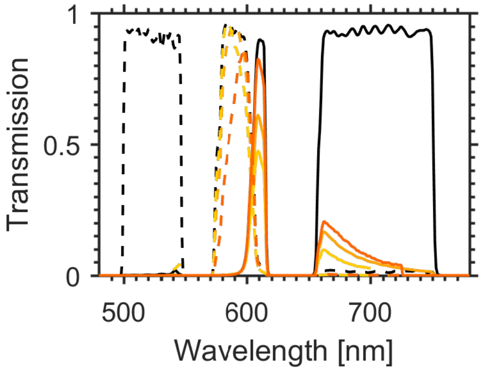

Supplementary Figure 9 Emission spectra of spectrally closely overlapping common organic fluorophores multiplied with the spectral response curve of the reflection and transmission channel obtained by a dichroic color splitter (~594 nm) and a multi-band dichroic and emission filter to suppress the excitation laser light. AbberiorFlip 565 (yellow), Atto 565 (orange), Alexa Fluor 568 (dark orange), spectral response of the reflection channel (black dashed line) and of the transmission channel (black solid line).

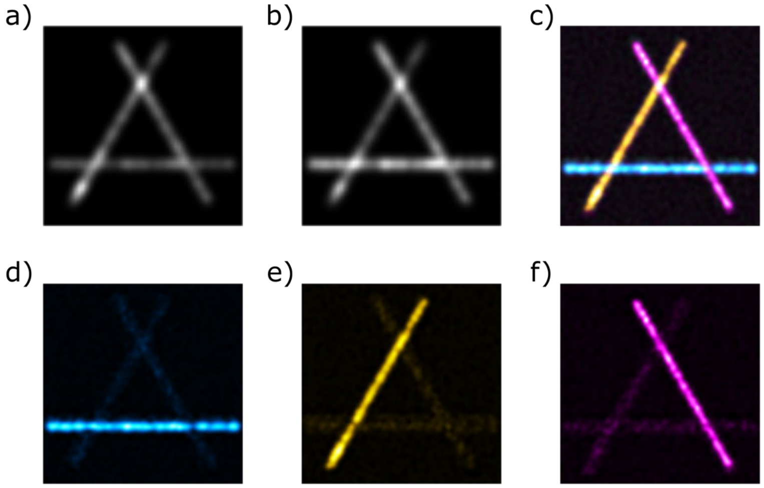

Supplementary Figure 10 Simulations of three fluorophores with spectrally very similar emission in the yellow to red range. Alexa Fluor 568 (horizontal filament), AbberiorFlip 565 (left, inclined to the right) and Atto 565 (right, inclined to the left) at a density of ~1000 fluorophores µm^{-2} for a 48 pixel × 0.4 pixel filament (corresponding to 5.184 µm × ~43 nm), I_{on} = 400 photons, on-ratio ρ_{on} = 0.1 (τ_{on} = 20 ms, τ_{off} = 180 ms) and τ_{PB} = 80 s. Channels were overlaid as simulated. a) and b) Average of 4000 frames in the reflection and transmission channel. c) RGB composite image of the unmixed and deconvolved second-order SOFI images with d) Alexa Fluor 568 (cyan hot), e) AbberiorFlip 565(yellow hot) and f) Atto 565 (magenta hot).

Fluorophore/channel	Alexa Fluor 568	Abberior Flip 565	Atto 565
**Alexa Fluor 568**	100	12	14
**Abberior Flip 565**	6	100	14
**Atto 565**	9	12	100

Supplementary Table 3 Relative crosstalk in % determined in the three colour simulated sample in Supplementary Figure 10 with the fluorophores Alexa Fluor 568, Abberior Flip 565 and Atto 565, when only background corrected pixels from regions without filament overlap were considered. The table is read line-wise with the fluorophore whose signal bleeds through listed on the left (e.g. read as: 6 % of Abberior Flip 565 is bleeding into the unmixed Alexa Fluor 568 channel).

Supplementary Note 6: Multicolor SOFI with spectral unmixing: additional data and resolution estimation

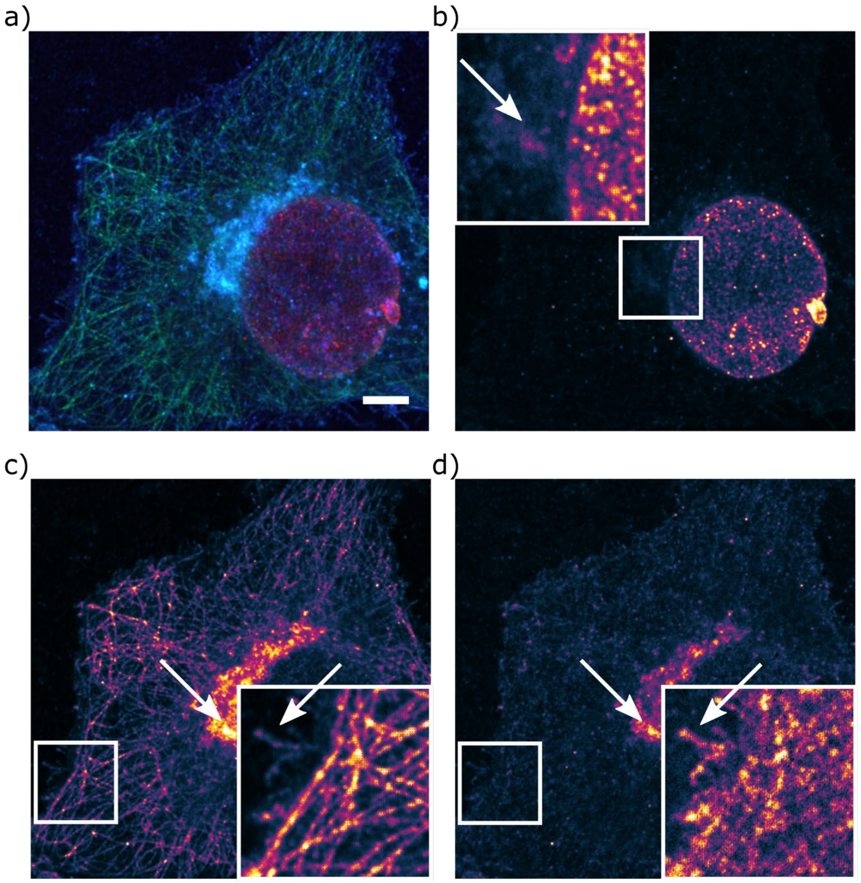

Supplementary Figure 11 Second order spectral cross-cumulant images of the cytoskeleton, nucleus and cellular membranes of HeLa cells. a) RGB composite image of the second order spectral cross-cumulant images with b) $\kappa_{2,TT}$(red), c) $\kappa_{2,RR}$(green) and d) $\kappa_{2,RT}$(blue). The separate cross-cumulant images are displayed using the morgenstemning colormap[7]. Scale bar 5 µm. Data from Figure 3. 200mM MEA with oxygen scavenging and about 0.5 kWcm^{-2} 488nm, 1.25 kWcm^{-2} 561nm and 1.3 kWcm^{-2} 635nm illumination intensity; 2000 frames and 20 ms exposure time. The arrows illustrate crosstalk of the fluorophores in the three spectral cross-cumulant channels. >10 images looked like this in at least two experiments that were performed.

Our multicolor approach is based on cumulant analysis and we used the same theoretical framework originally devised for spatially super-resolved SOFI[1]. The theoretically attainable resolution increase for second order analysis with respect to widefield imaging is two-fold after spectral cross-cumulation and post-processing (using deconvolution and linearization[2] (used here) or Fourier reweighing[3]).

We estimated the resolution of our imaging using our implementation of image decorrelation analysis in ImageJ (default settings)[8]. This approach uses partial phase autocorrelation for a series of filtered images to determine the highest spatial frequency with sufficiently high signal in relation to noise. For the fixed cells in Figure 3 (microtubules-Alexa488 λ_{em}= 519 nm, WGA-Atto565 λ_{em}= 592 nm and Lamin B1-Alexa647 λ_{em}= 665 nm), the smallest features are 317 nm in the reflected channel I_R and 325nm in the transmitted channel I_T (average image of the time series). Both channels contain signals from all three dyes, albeit at different proportions, and camera noise as well as out of-focus signal.

The theoretical widefield resolution of ideal point sources according to the Abbe criterion $d = \frac{\lambda_{em}}{2NA}$ (NA=1.27) is estimated at the maximum fluorescence emission as 204 nm, 233 nm and 262 nm for the three dyes (disregarding the fluorescence emission tail at longer wavelengths); the corresponding theoretical SOFI resolution is twofold improved 102 nm, 117 nm and 131 nm. Taking into account the underlying structure of e.g. microtubule apparent diameter of 25 nm + 40 nm (secondary immunostaining increases the imaged microtubule diameter by 20-40 nm4), the expected theoretical resolution in the absence of noise e.g. in the Alexa488 unmixed SOFI channel is $\sqrt{65^2 + 102^2}\, nm = 121\, nm.$

After deconvolution and linearization, we estimate 151 nm in the Alexa488 channel (2.09 fold improvement vs. I_R), 154 nm in the Atto565 channel (2.06/2.11 fold improvement vs. $I_{R/T}$) and 159 nm in the Alexa647 channel (2.04 fold improvement vs. I_T) for the results shown in Figure 3c-f. The estimations and fold improvement are reasonable considering the finite resolution assessment accuracy and considering that the widefield images contain a mixture of the three fluorophores and background fluorescence.

Supplementary Note 7: Multicolor SOFI with spectral unmixing: additional experiments

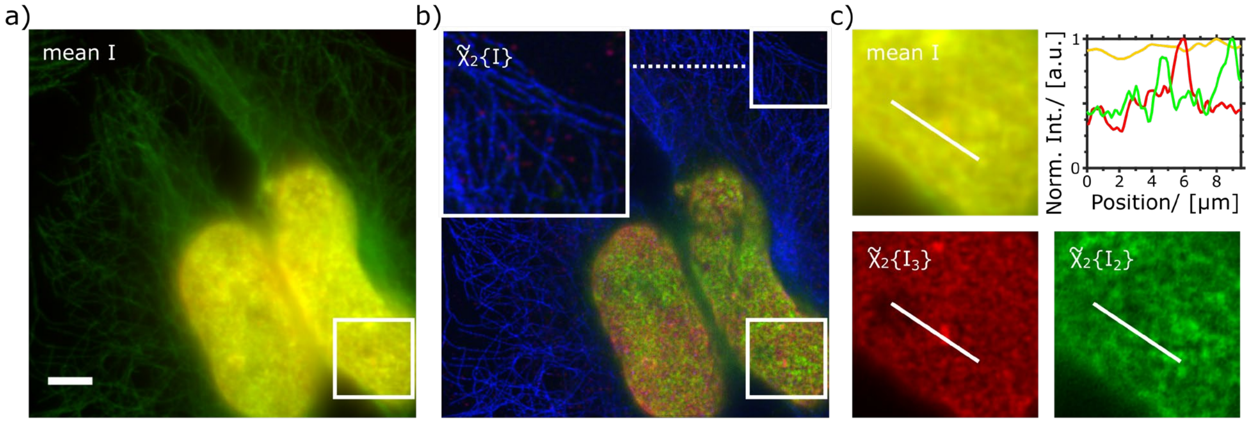

Supplementary Figure 12 Multicolor SOFI of the cytoskeleton and nucleus of HeLa cells.a) Overlay of the average of the time series acquired in the reflection (green) and transmission (red) channel using 50 mM MEA with oxygen scavenging and about 0.5 kWcm^{-2} 488 nm , 1.25 kWcm^{-2} 561 nm and 1.3 kWcm^{-2} 635 nm illumination intensity; 2000 frames and 20 ms exposure time. b) RGB composite image of the unmixed, flattened and deconvolved second order SOFI images with Alexa Fluor 488 secondary antibody stained microtubules (blue, $\tilde{\chi}_2\{I_1\}$), Hoechst-Janelia Fluor 549 DNA labeling (green, $\tilde{\chi}_2\{I_2\}$) and Alexa Fluor 647 secondary antibody stained nuclear membrane (red, $\tilde{\chi}_2\{I_3\}$). c) Close-up of the ROI indicated in a) and b), respectively and comparison of the normalized intensity profiles along the indicated line. Scale bar 5 µm. >5 images looked like this in at least two experiments that were performed.

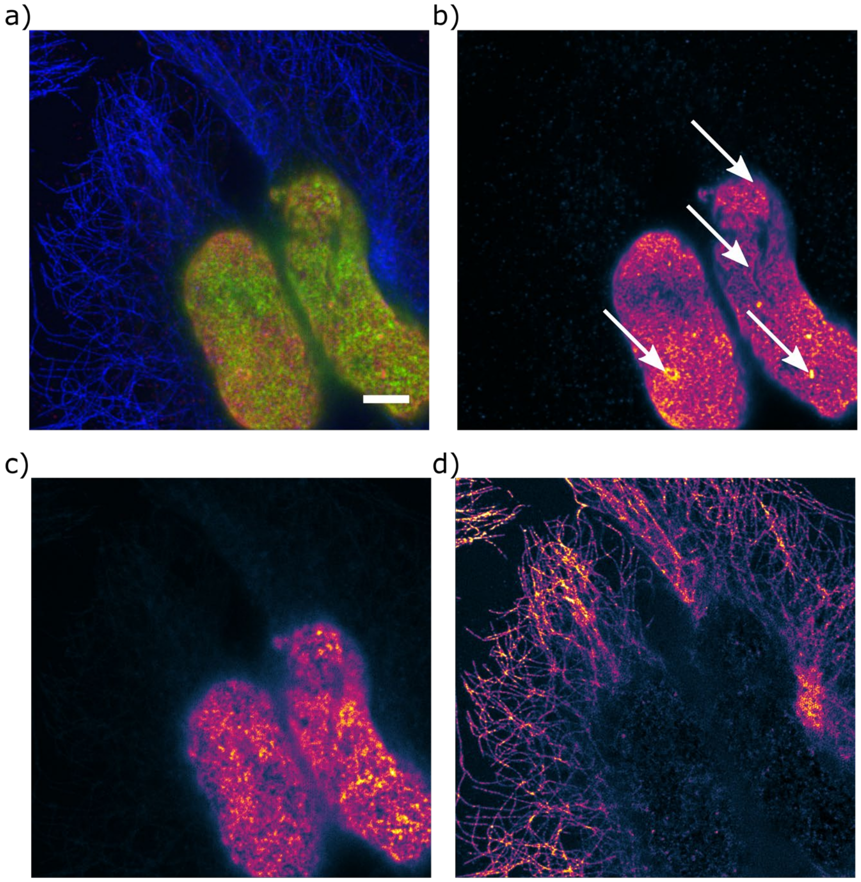

Supplementary Figure 13 Multicolor SOFI of the cytoskeleton and nucleus of HeLa cells.a) RGB composite image of the unmixed, flattened and deconvolved second order SOFI images with b) Alexa Fluor 647 secondary antibody stained nuclear membrane (red), c) Hoechst-Janelia Fluor 549 DNA labeling (green) and. d) Alexa Fluor 488 secondary antibody stained microtubules (blue). The separate unmixed images are displayed using the morgenstemning colormap[7]. The arrows in b) indicate typical features of Lamin B staining such as folds in the nuclear membrane. Scale bar 5 µm. Data from Figure 4. 50mM MEA with oxygen scavenging and about 0.5 kWcm^{-2} 488nm, 1.25 kWcm^{-2} 561nm and 1.3 kWcm^{-2} 635nm illumination intensity; 2000 frames and 20 ms exposure time. >5 images looked like this in at least two experiments that were performed.

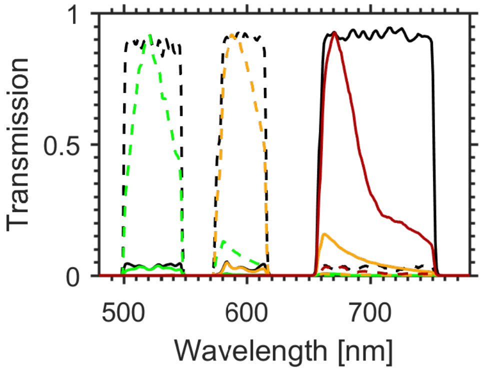

Supplementary Figure S14 Emission spectra of common organic fluorophores multiplied with the spectral response curve of the reflection and transmission channel obtained by a dichroic color splitter (~640 nm) and a multi-band dichroic and emission filter to suppress the excitation laser light. Alexa Fluor 488 (green), Atto 565 (orange), Alexa Fluor 647 (red), spectral response of the reflection channel (black dashed line) and of the transmission channel (black solid line).

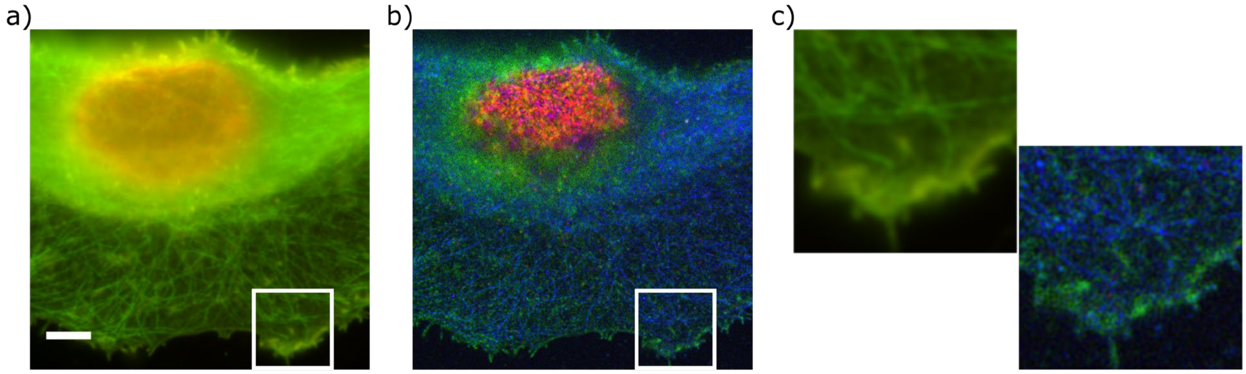

Supplementary Figure 15 Multicolor SOFI of the cytoskeleton, nucleus and cellular membranes of HeLa cells with a dichroic splitting ~640 nm. a) Overlay of the average of the time series acquired in the reflection (green) and transmission channel (red) using 200mM MEA with oxygen scavenging and about 0.5 kWcm^{-2} 488nm, 1.25 kWcm^{-2} 561nm and 0.85 kWcm^{-2} 635nm illumination intensity; 2000 frames and 20 ms exposure time. b) RGB composite image of the unmixed and deconvolved second order SOFI images with Alexa Fluor 488 secondary antibody stained microtubules (blue), wheat germ agglutinin-Atto565 labeling (green) and Alexa Fluor 647 secondary antibody stained nuclear membrane (red). c) Close-up of the ROI indicated in a) and b), respectively. Scale bar 5 µm. >3 images looked like this in at least two experiments that were performed.

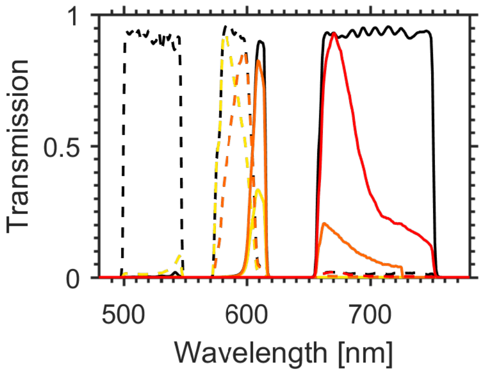

Supplementary Figure 16 Emission spectra of common organic fluorophores multiplied with the spectral response curve of the reflection and transmission channel obtained by a dichroic color splitter (~594 nm) and a multi-band dichroic and emission filter to suppress the excitation laser light. Janelia Fluor 549 (yellow), Alexa Fluor 568 (dark orange), Alexa Fluor 647 (red), spectral response of the reflection channel (black dashed line) and of the transmission channel (black solid line).

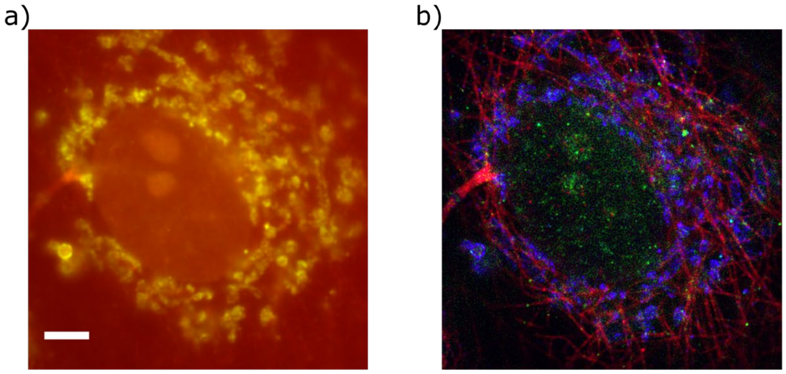

Supplementary Figure 17 Multicolor SOFI of the cytoskeleton, nucleus and mitochondria of COS-7 cells with a dichroic splitting ~594 nm. a) Overlay of the average of the time series acquired in the reflection (green) and transmission channel (red) using 50mM MEA with oxygen scavenging and about 1.25 kWcm^{-2} 561nm and 1.3 kWcm^{-2} 635nm illumination intensity; 2000 frames and 20 ms exposure time. b) RGB composite image of the unmixed and deconvolved second order SOFI images with Alexa Fluor 568 secondary antibody stained mitochondria (blue), Hoechst-Janelia Fluor 549 labeling (green) and Alexa Fluor 647 secondary antibody stained microtubules (red). Scale bar 5 µm. The staining of the DNA is sparse and was performed with 5 times lower concentration as for HeLa cells shown in the main text. >3 images looked like this in the experiment that was performed.

Supplementary Note 8: Multicolor SOFI with spectral unmixing: additional data live cell experiments

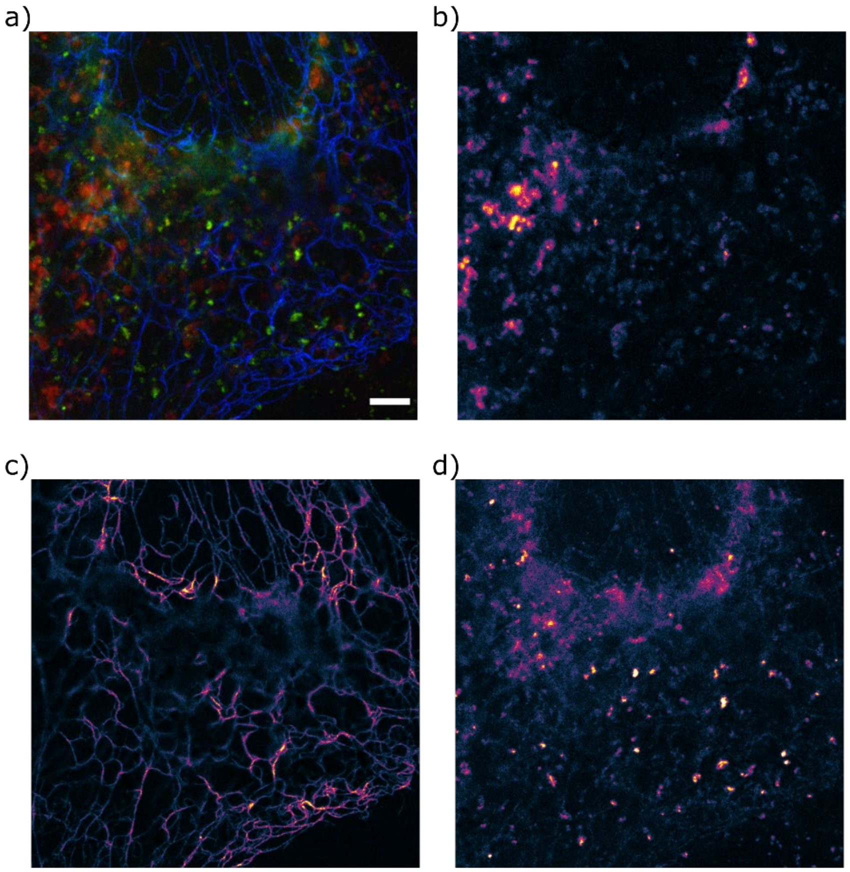

Supplementary Figure 18 Multicolor SOFI live cell imaging of the cytoskeleton, nucleus and wheat-germ agglutinin in COS-7 cells. a) RGB composite image of the unmixed, flattened and deconvolved SOFI images with b) Mitotracker Deep Red FM stained mitochondria (red, $\tilde{\chi}_2\{I_3\}$,) c) Vimentin-Dreiklang fluorescent protein expression (blue, $\tilde{\chi}_2\{I_1\}$) and d) accumulated wheat germ agglutinin-AbberiorFlip565 labeling (green, $\tilde{\chi}_2\{I_2\}$). Data from Figure 4. Scale bar 5 µm, all displayed using the morgenstemning colormap[27]. >3 images looked like this in the experiment that was performed.

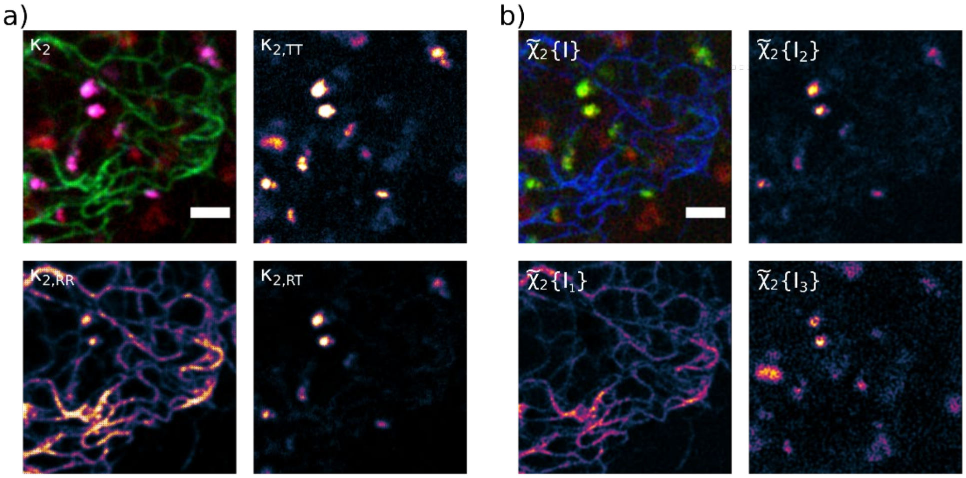

Supplementary Figure 19 Multicolor SOFI live cell imaging; spectral cross-cumulants and unmixed, flattened and deconvolved SOFI a) RGB composite image of the second order spectral cross-cumulant images with $\kappa_{2,RR}$(green), $\kappa_{2,RT}$(blue) and $\kappa_{2,TT}$(red) and single channel images of the ROIs indicated in Figure 4 a) and b). b) RGB composite image of the unmixed, flattened and deconvolved SOFI images with Mitotracker Deep Red FM stained mitochondria (brightness enhanced, red, $\tilde{\chi}_2\{I_3\}$), accumulated wheat germ agglutinin-AbberiorFlip565 labeling (green, $\tilde{\chi}_2\{I_2\}$) and Vimentin-Dreiklang fluorescent protein expression (blue, $\tilde{\chi}_2\{I_1\}$) and single channel images of the ROIs indicated in Figure 4 a) and b). Scale bars 2 µm. Single channel images are displayed using the morgenstemning colormap[27]. Scale bars 2 µm. >3 images looked like this in the experiment that was performed.

References

1. Dertinger, T., Colyer, R., Iyer, G., Weiss, S. & Enderlein, J. Fast, background-free, 3D super-resolution optical fluctuation imaging (SOFI). *Proc. Natl. Acad. Sci. U.S.A.* **106,** 22287–22292 (2009).
2. Geissbuehler, S. *et al.* Mapping molecular statistics with balanced super-resolution optical fluctuation imaging (bSOFI). *Opt Nanoscopy* **1,** 1–7 (2012).
3. Dertinger, T., Colyer, R., Vogel, R., Enderlein, J. & Weiss, S. Achieving increased resolution and more pixels with Superresolution Optical Fluctuation Imaging (SOFI). *Opt Express* **18,** 18875–18885 (2010).
4. Vandenberg, W. *et al.* Model-free uncertainty estimation in stochastical optical fluctuation imaging (SOFI) leads to a doubled temporal resolution. *Biomed Opt Express* **7,** 467–14 (2016).
5. Girsault, A. *et al.* SOFI Simulation Tool: A Software Package for Simulating and Testing Super-Resolution Optical Fluctuation Imaging. *PLoS ONE* **11,** e0161602–13 (2016).
6. Dempsey, G. T., Vaughan, J. C., Chen, K. H., Bates, M. & Zhuang, X. Evaluation of fluorophores for optimal performance in localization-based super-resolution imaging. *Nat Methods* **8,** 1027–1036 (2011).
7. Geissbuehler, M. & Lasser, T. How to display data by color schemes compatible with red-green color perception deficiencies. *Opt Express* **21,** 9862–13 (2013).
8. Descloux, A., Grussmayer, K. S. & Radenovic, A. Parameter-free image resolution estimation based on decorrelation analysis. Nat Methods **16**, 918–924(2019).
