# Supplementary material for: Spectral cross-cumulants for multicolor super-resolved SOFI imaging
Source: Nat Commun. 2020 Jun 15;11:3023. doi: 10.1038/s41467-020-16841-1 (PMC7295763; doi:10.1038/s41467-020-16841-1)
Supplement: Supplementary file 3 — Reporting Summary [file 41467_2020_16841_MOESM3_ESM.pdf]

naturere search

Corresponding author(s): Kristin Grussmayer, Theo Lasser

Last updated by author(s): 04.05.2020

# Reporting Summary

Nature Research wishes to improve the reproducibility of the work that we publish. This form provides structure for consistency and transparency in reporting. For further information on Nature Research policies, see Authors & Referees and the Editorial Policy Checklist .

## Statistics

For all statistical analyses, confirm that the following items are present in the figure legend, table legend, main text, or Methods section.

| n/a | Confirmed | |
|---|---|---|
| ☐ | ☒ | The exact sample size ($n$) for each experimental group/condition, given as a discrete number and unit of measurement |
| ☐ | ☒ | A statement on whether measurements were taken from distinct samples or whether the same sample was measured repeatedly |
| ☒ | ☐ | The statistical test(s) used AND whether they are one- or two-sided<br>*Only common tests should be described solely by name; describe more complex techniques in the Methods section.* |
| ☒ | ☐ | A description of all covariates tested |
| ☒ | ☐ | A description of any assumptions or corrections, such as tests of normality and adjustment for multiple comparisons |
| ☒ | ☐ | A full description of the statistical parameters including central tendency (e.g. means) or other basic estimates (e.g. regression coefficient) AND variation (e.g. standard deviation) or associated estimates of uncertainty (e.g. confidence intervals) |
| ☒ | ☐ | For null hypothesis testing, the test statistic (e.g. $F$, $t$, $r$) with confidence intervals, effect sizes, degrees of freedom and $P$ value noted<br>*Give P values as exact values whenever suitable.* |
| ☒ | ☐ | For Bayesian analysis, information on the choice of priors and Markov chain Monte Carlo settings |
| ☒ | ☐ | For hierarchical and complex designs, identification of the appropriate level for tests and full reporting of outcomes |
| ☒ | ☐ | Estimates of effect sizes (e.g. Cohen's $d$, Pearson's $r$), indicating how they were calculated |

*Our web collection on statistics for biologists contains articles on many of the points above.*

## Software and code

Policy information about availability of computer code

| | |
|---|---|
| Data collection | Microscopy data was collected using MicroManager 1.4 using available plugins/core functionality for the cameras and stage and a bean shell script; a custom plugin was written to control shutters for lasers via an Arduino. |
| Data analysis | We used custom code written in Matlab (we describe the code workflow in the manuscript and provide the source code including instructions) and used Matlab R2016b under Windows 7 for analysis. |

For manuscripts utilizing custom algorithms or software that are central to the research but not yet described in published literature, software must be made available to editors/reviewers. We strongly encourage code deposition in a community repository (e.g. GitHub). See the Nature Research guidelines for submitting code & software for further information.

## Data

Policy information about availability of data

All manuscripts must include a data availability statement. This statement should provide the following information, where applicable:

- Accession codes, unique identifiers, or web links for publicly available datasets
- A list of figures that have associated raw data
- A description of any restrictions on data availability

All data needed to evaluate the conclusions in the paper are present in the paper and/or the Supplementary Materials. All relevant raw data are available from the authors upon reasonable request.

# Field-specific reporting

Please select the one below that is the best fit for your research. If you are not sure, read the appropriate sections before making your selection.



October 2018

# Life sciences study design

All studies must disclose on these points even when the disclosure is negative.

| | |
|---|---|
| Sample size | We were not performing a biological study with statistical analysis; we show examples of successful imaging with triple labeled cells. We optimized staining by titration of antibody or amount of transfection reagent:plasmid DNA for single labeled cells first. Subsequently, we achieved consistent results of triple labeled cells across typically > 3-10 different imaged cells for a certain staining and considered this sufficient to ensure reproducibility. For fixed cell imaging, the views were representative of the whole imaging chamber; for live cell imaging there was a big variation in protein expression and we selected cells representative for low-medium Vimentin-Dreiklang expression. |
| Data exclusions | N/a |
| Replication | We achieved consistent results of triple labeled cells across > 3-10 different imaged cells for a certain staining; typically the staining and imaging was repeated in at least two independent experiments. |
| Randomization | N/A |
| Blinding | N/A |

# Reporting for specific materials, systems and methods

We require information from authors about some types of materials, experimental systems and methods used in many studies. Here, indicate whether each material, system or method listed is relevant to your study. If you are not sure if a list item applies to your research, read the appropriate section before selecting a response.

## Materials & experimental systems

| n/a | Involved in the study | |
|---|---|---|
| ☐ | ☒ | Antibodies |
| ☐ | ☒ | Eukaryotic cell lines |
| ☒ | ☐ | Palaeontology |
| ☒ | ☐ | Animals and other organisms |
| ☒ | ☐ | Human research participants |
| ☒ | ☐ | Clinical data |

## Methods

| n/a | Involved in the study | |
|---|---|---|
| ☒ | ☐ | ChIP-seq |
| ☒ | ☐ | Flow cytometry |
| ☒ | ☐ | MRI-based neuroimaging |

## Antibodies

| | |
|---|---|
| Antibodies used | anti alpha-tubulin antibody (dilution 1:150, clone [DM1a] mouse monoclonal, ab7291, Lot GR310199-6, Abcam); anti alpha-tubulin antibody (dilution 1:100-1:200, clone B-5-1-2 mouse monoclonal ascites fluid, T5168 , Lot 047M4760V, Sigma-Aldrich) ; donkey anti-Mouse IgG (H+L) Highly Cross-Adsorbed Secondary Antibody, Alexa Fluor 647 (0.005 mg ml-1, provided by histology core facility (EPFL), A-31571, Invitrogen); donkey anti-Rabbit IgG (H+L) Highly Cross-Adsorbed Secondary Antibody, Alexa Fluor 488 (0.01 mg ml-1, provided by histology core facility (EPFL), A-21206, Invitrogen); donkey anti-Rabbit IgG (H+L) Highly Cross-Adsorbed Secondary Antibody, Alexa Fluor 568 (0.005 mg ml-1, provided by histology core facility (EPFL), A-10042, Invitrogen); anti-Lamin B1 antibody (dilution 1:400, rabbit polyclonal, ab16048 , Abcam); anti-TOMM20 antibody (1:50 dilution, clone [EPR15581], ab186734, Abcam) |
| Validation | anti alpha-tubulin antibody (clone [DM1a] mouse monoclonal, ab7291, Abcam) used in HeLa (Human) cells. The manufacture's website shows validation that the antibody is specific for Homo sapiens (Human) protein and is showing successful immunofluorescence in HeLa cells. The antibody is highly cited for immunofluorescence according to citeab.com.<br><br>anti-Lamin B1 antibody (rabbit polyclonal, ab16048 , Abcam) used in HeLa (Human) cells. The manufacture's website shows validation that the antibody is specific for Homo sapiens (Human) protein and is showing successful immunofluorescence in HeLa cells. The antibody is highly cited for immunofluorescence according to citeab.com.<br><br>anti alpha-tubulin antibody (clone B-5-1-2 mouse monoclonal ascites fluid, T5168 , Sigma-Aldrich) used in COS-7 (African green monkey) cells. The manufacture's website shows validation that the antibody is specific for African green monkey protein. The antibody is highly cited for immunofluorescence according to citeab.com. |

## Eukaryotic cell lines

Policy information about cell lines

| | |
|---|---|
| Cell line source(s) | HeLa cells are from ATCC, COS-7 cells were a kind gift of the Manley lab (EPFL) |
| Authentication | none of the cell lines used were authenticated |

| | |
|---|---|
| Mycoplasma contamination | cell lines were not tested for Mycoplasma contamination |
| Commonly misidentified lines<br>(See ICLAC register) | no commonly misidentified cell lines were used |
